# PCAF fine‐tunes hepatic metabolic syndrome, inflammatory disease, and cancer

**DOI:** 10.1111/jcmm.13877

**Published:** 2018-09-14

**Authors:** Tongxin Wang, Weilei Yao, Yafei Shao, Ruilong Zheng, Feiruo Huang

**Affiliations:** ^1^ Department of Animal Nutrition and Feed Science College of Animal Science and Technology Huazhong Agricultural University Wuhan China

**Keywords:** cancer, fine‐tuning, hepatic metabolic syndrome, inflammatory disease, PCAF

## Abstract

The P300/CBP‐associating factor (PCAF), a histone acetyltransferase, is involved in metabolic and pathogenic diseases, particularly of the liver. The effects of PCAF on fine‐tuning liver diseases are extremely complex and vary according to different pathological conditions. This enzyme has dichotomous functions, depending on differently modified sites, which regulate the activities of various enzymes, metabolic functions, and gene expression. Here, we summarize the most recent findings on the functions and targets of PCAF in various metabolic and immunological processes in the liver and review these new discoveries and models of PCAF biology in three areas: hepatic metabolic syndrome, inflammatory disease, and cancer. Finally, we discuss the potential implications of these findings for therapeutic interventions in liver diseases.

## 1 INTRODUCTION

Obesity, type 2 diabetes mellitus (T2DM), and other disturbances of chronic metabolic syndrome are now worldwide health problems.^1^, ^2^, ^3^ There is a parallel trend of the incidence and prevalence of immune diseases and tumorigenesis.^4^, ^5^ The liver, the largest visceral organ in the body, has been intimately associated with various diseases including chronic metabolic syndrome, inflammation, and cancer.^6^, ^7^, ^8^, ^9^ Under pathological conditions, the activation of immune cells and inflammatory pathways lead to liver complications, including damage to hepatic metabolic homeostasis and a cluster of interrelated metabolic risk factors such as raised fasting glucose, central obesity, dyslipoproteinaemia, and hypertension.^10^, ^11^, ^12^, ^13^ In addition, the damage of hepatic metabolic homeostasis and inflammatory disease has gained increasing attention for its relationship with end‐stage liver disease: primary liver cancer and hepatocellular carcinoma (HCC).^14^, ^15^, ^16^

Several transcription factors are concurrently critical mediators in the regulation of biological processes, especially under illness stress.^17^, ^18^ These transcription factors are subjected to posttranslational modifications (PTMs) that affect their activity, stability, intracellular distribution, and interaction with other proteins.^19^, ^20^, ^21^, ^22^, ^23^ During the past decades, the inventory of acetylation, demonstrated by the number of modification sites, is fast catching up with other major PTMs, such as phosphorylation and ubiquitylation, highlighting the regulatory potential of this modification at the proteomics scale.^24^ Recent studies have reported that overall cellular metabolism can be regulated through acetylation.^25^, ^26^, ^27^, ^28^ Predictably, as a pivotal metabolic organ, the liver is largely subjected to lysine acetylation, which occurs in most of the metabolic enzymes in human liver cells involved in glycolysis, tricarboxylic acid (TCA) and urea cycles, and fatty acid and glycogen metabolism.^29^, ^30^, ^31^, ^32^, ^33^ The acetylation status of these enzymes may alter their activities in order to respond to any changes in metabolic pathological demands.^34^, ^35^

The P300/CBP‐associating factor (PCAF) is a histone acetyltransferase (HAT) that primarily acetylates H3 histones and has a strong association with tumour initiation and progression, and it is similar to other HAT family member‐GCN5 eukaryotes.^36^ Using various nutritional, genetic, and pharmacological model systems, the roster of PCAF‐acetylated lysine sites has rapidly expanded. An emergent theory indicates that PCAF is also involved in multiple hepatic metabolic and pathogenic diseases such as metabolic syndrome, inflammation, apoptosis, injury, and cancer. For example, PCAF can acetylate nonhistone proteins, including, phosphoglycerate kinase 1 (PGK1) (K323), ATP‐citrate lyase (ACLY) (K540, K546, and K554), Peroxisome proliferator‐activated receptor gamma coactivator 1‐alpha (PGC1‐α) (K328 and K450), forkhead box P3 (FOXP3), and p53, etc.^37^, ^38^, ^39^, ^40^, ^41^ Additionally, increasing evidence demonstrates that PCAF is not only a HAT but also shows other effects such as ubiquitination.^42^ This review primarily focuses on the different mechanisms by which PCAF fine‐tunes hepatic metabolic syndrome, inflammatory disease, and tumour growth.

## 2 PCAF IN HEPATIC METABOLIC SYNDROME

As an associated factor of p300/CBP, PCAF and has been identified based on its activity in histone acetylation and is implicated in various cellular processes including proliferation and apoptosis.^43^, ^44^ More recently, a growing body of evidence has implicated PCAF in hepatic metabolic homeostasis, including lipogenesis, fatty acid oxidation, gluconeogenesis, and the regulation of insulin action, suggesting that PCAF activity may ensure the coordinated regulation of several distinct metabolic functions in the liver. Further study of PCAF will likely contribute to the development of treatments for hepatic metabolic syndrome and other nonalcoholic fatty liver diseases.

### 2.1 Hepatic lipid metabolism

#### 2.1.1 Hepatic de novo lipogenesis and hyperlipidaemia

**Figure 1:**
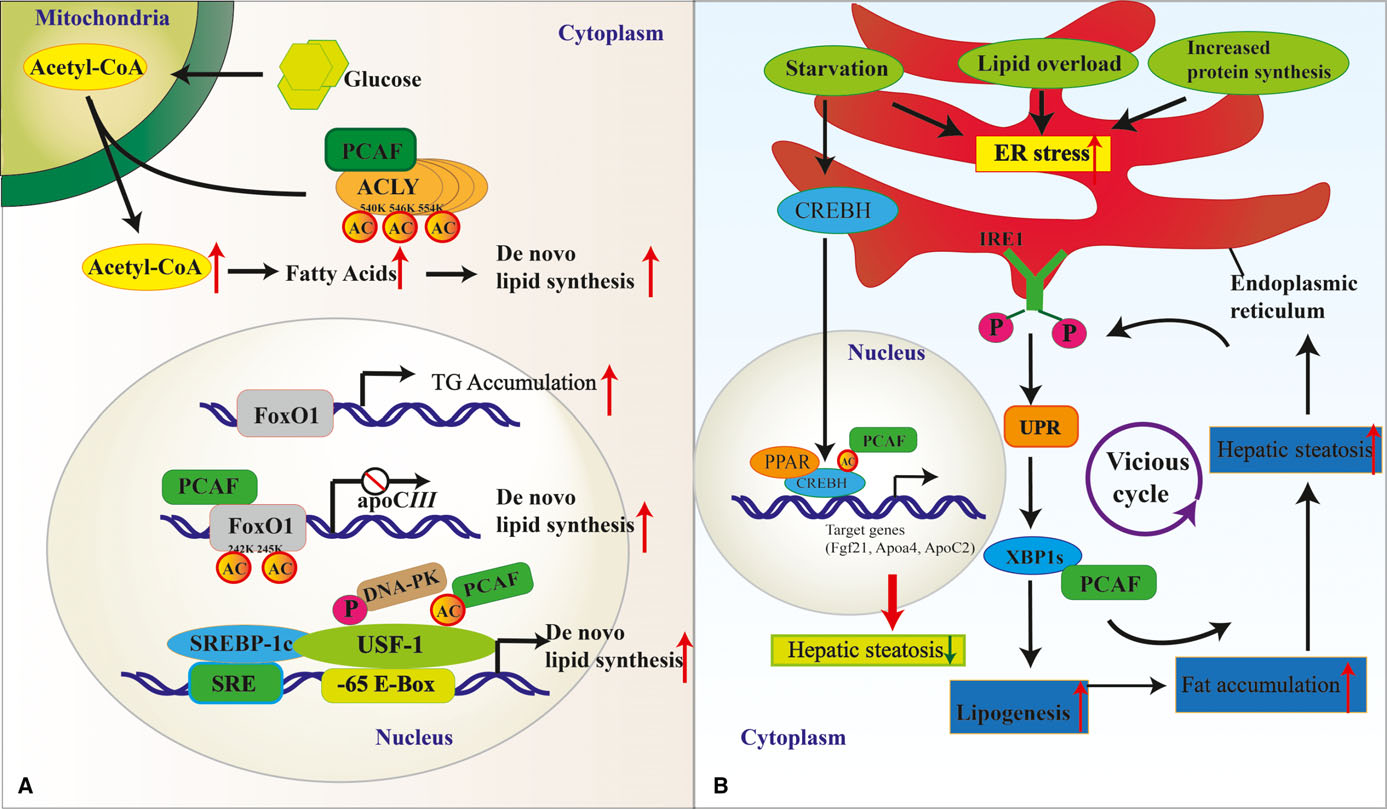
Potential contributions of PCAF‐induced acetylation to the development of hepatic lipid metabolism. A, Hepatic de novo lipogenesis and hyperlipidaemia in PCAF regulation: the scheme indicates the up‐ and downstream signalling regulatory actions of the inducible PCAF acetylation and their effects on de novo lipogenesis both in enzymatic and transcriptional events. B, Nonalcoholic fatty liver disease and ER stress in PCAF regulation: lipid overboard, starvation, increased protein synthesis will lead to ER stress. The scheme indicates the enzyme and transcription factors that regulate ER stress and hepatic steatosis controlled by PCAF. PCAF has both positive and negative effects on occurrence of hepatic steatosis in different pathways. ACLY, ATP‐citrate lyase; FoxO1, forkhead box O1; USF‐1, upstream stimulatory factor‐1; SREBP‐1c, sterol regulatory element‐binding protein‐1c; DNA‐PK, DNA‐dependent protein kinase; apoC‐III, apolipoprotein C‐III; UPR, unfolded protein response; XBP‐1, X‐box‐binding protein1; AC, acetylation; ER, endoplasmic reticulum

Hyperlipidaemia is a prevalent disease and a major component of metabolic syndromes.^45^, ^46^ In a physiological study, using a diabetic pregnant rat model, Abraham and colleagues showed that increased hepatic de novo lipogenesis directly contributes to hyperglycaemia and hyperlipidaemia.^47^ Apolipoprotein apoC‐III is important in lipogenesis and triglyceride (TG) metabolism. Hepatic apoC‐III production is subject to insulin inhibition, and is effectively up‐regulated by forkhead box O1 (FoxO1). A previous study on mice demonstrated that FoxO1 deregulation is associated with insulin deficiency or insulin resistance. Elevated FoxO1 production in the liver augments hepatic apoC‐III expression, resulting in increased plasma TG levels and impaired fat tolerance.^48^ In addition, transgenic mice expressing a constitutively nuclear FoxO1 allele directly exhibited hypertriglyceridaemia.^48^ These results provide several lines of evidence that FoxO1 functions as a regulator of lipogenesis and TG metabolism through different pathways. Remarkably, Yoshimochi et al recently showed that PCAF repressed FoxO1‐induced transcription in an enzymatic activity‐independent manner.^49^ PCAF bound to the forkhead domain of FoxO1 and acetylated FoxO1 at the K242 and K245 residues, while Akt‐induced phosphorylation of FoxO1 is required for its binding to PCAF, and this binding inhibits FoxO1‐induced transcription in the nucleus.^49^ Hence, the binding between PCAF and FoxO1 reduces lipid accumulation via the inhibition of TG synthesis (Figure 1A), thus providing a novel therapeutic strategy for the treatment of FoxO1‐induced diabetic hypertriglyceridaemia.

Fatty acid synthase (FAS) is another key enzyme in hepatic de novo lipogenesis. It is also transcriptionally activated in response to feeding and insulin signalling.^50^ Two decades ago, Moustaïd et al showed that binding of the upstream stimulatory factor‐1/2 (USF‐1/2) heterodimer to the ‐65 E‐box is required for FAS promoter activation.^51^, ^52^, ^53^ Additionally, several trait‐mapping studies in humans have identified USF‐1 as a candidate gene for familial combined hyperlipidemia.^54^ Recently, Wong and Sul showed that during fasting USF‐1 recruits HDAC9, which deacetylates USF‐1 to repress transcription despite its binding to the ‐65 E‐box.^55^ Intriguingly, after feeding, the DNA‐dependent protein kinase (DNA‐PK) phosphorylates USF‐1, which subsequently recruits SREBP‐1c and PCAF and is acetylated at K237. This binding directly results in FAS promoter activation in the liver and subsequently promotes lipogenesis. In their study, K237 acetylation is dependent on S262 phosphorylation in response to feeding/insulin via preferential interaction with PCAF.^55^ Thus, the phosphorylation‐dependent acetylation of USF‐1 by PCAF and DNA‐PK functions as a dynamic molecular switch in sensing the nutritional transition from fasting to feeding. Such a multistep switch provides a mechanism to fine‐tune the transcription of lipogenic genes in response to different nutritional states (Figure 1A).

#### 2.1.2 Nonalcoholic fatty liver disease and endoplasmic reticulum stress

The long‐term state of hyperlipidaemia leads to liver pathologies referred to as nonalcoholic fatty liver disease (NAFLD), which represents a large spectrum of diseases, including hepatic steatosis, fatty liver, and nonalcoholic steato hepatitis (NASH).^56^, ^57^, ^58^, ^59^ Deciphering the specific activities and substrates of PCAF mediating its different physiological functions in NAFLD has become an important area of research, as these functions may not only be limited to acetylation. According to one previous study, Lew et al showed that PCAF mediates X‐box‐binding protein1 (XBP‐1)‐dependent transcription through interaction with XBP‐1S. XBP‐1S is the necessary transcriptional activator of unfolded protein response, which is triggered when the endoplasmic reticulum (ER) is under stress.^60^ ER stress has been closely associated with hepatic steatosis, as hepatic lipid overload has been implicated in the initiation of the chronic ER stress in steatosis.^61^, ^62^ Steatosis is the first step in the progression of NAFLD, and is characterized by lipid accumulation in hepatocytes and is highly prevalent in people with obesity and hyperlipidaemia.^63^, ^64^ The PCAF‐induced translation of the spliced XBP1 mRNA generates a very potent form of the XBP1 transcription factor, which in turn increases the expression of ER chaperones and ER‐associated degradation under stress.^65^ Although the liver only undergoes transient ER stress under physiological conditions, this ER stress becomes chronic in NAFLD, as demonstrated by the detection of ER stress responses in the fatty liver.^66^ Therefore, these studies indicate that PCAF‐binding XBP1 is involved in activating ER stress‐induced hepatic steatosis/fatty liver (Figure 1B).

Cyclic AMP‐responsive element‐binding protein‐like 3(CREBH) is a hepatocyte‐specific ER‐anchored transcription factor involved in the initiation of NAFLD.^67^ Numerous evidence indicates that CREBH functions as a key regulator of energy homeostasis.^68^, ^69^, ^70^ Kim and colleagues observed that CREBH interacts with Peroxisome proliferator‐activated receptor alpha (PPARα) to synergistically activate the metabolic hormone fibroblast growth factor 21 to regulate lipolysis, fatty acid oxidation, and ketogenesis upon fasting or under an atherogenic high‐fat diet.^69^ Similar results were reported in another study using metabolic stress mice: defects in CREBH directly lead to nonalcoholic steatohepatitis and hyperlipidaemia under an atherogenic high‐fat diet or fasting conditions.^68^, ^70^ More recently, an emerging research by Kim demonstrated that PCAF and SIRT1 are the acetyltransferase and deacetylase of CREBH, respectively, under fasting conditions. CREBH is acetylated at lysine 294 within the CREBH bZIP domain by PCAF. Acetylation at this site is required for CREBH transcriptional activity.^71^ PCAF‐mediated acetylation and the transcriptional activities of CREBH are required for the interaction and synergy between CREBH and PPARα in activating their target gene upon fasting (Figure 1B). CREBH acetylation at lysine 294 is critical to maintain hepatic lipid homeostasis in fasting states.

Considering that ER stress promotes fat accumulation in hepatocytes and induces NAFLD, Baiceanu et al reported that this condition primarily occurs via the induction of de novo lipogenesis.^72^ ACLY is a lipogenic enzyme that catalyses the conversion of cytosolic citrate to acetyl‐CoA, which is the building block for de novo lipid synthesis.^73^ A recent study provided evidence that under high‐glucose conditions, ACLY is acetylated in both cells and mouse liver by PCAF acetyltransferase, which increases its stability and promotes de novo lipid synthesis.^38^ This finding has important implications in the context of metabolic regulation, as PCAF‐dependent ACLY acetylation could provide a strategy to improve lipogenesis (Figure 1B). This observation is consistent with the results of a previous study in which liver‐specific ACLY down‐regulation in leptin receptor‐deficient db/db mice led to the inhibition of hepatic de novo lipogenesis and protection against hepatic steatosis.^74^ Therefore, in this case, approaches to reduce hepatic PCAF activity or expression could serve as potential therapeutic strategies for the treatment of fatty liver disease.

### 2.2 Hepatic glucose homeostasis

#### 2.2.1 PGC1‐α and Foxo1 acetylation in hepatic gluconeogenesis

In 1898, Naunyn coined the term hepatogenous diabetes to describe the coincidence between diabetes and liver glucose disorder, emphasizing the pathological importance of the liver in regulating glucose metabolism.^75^ Several transcription factors and co‐activators are involved in the nutritional and hormonal control of gluconeogenesis, including PGC‐1α and FoxO1.

**Figure 2:**
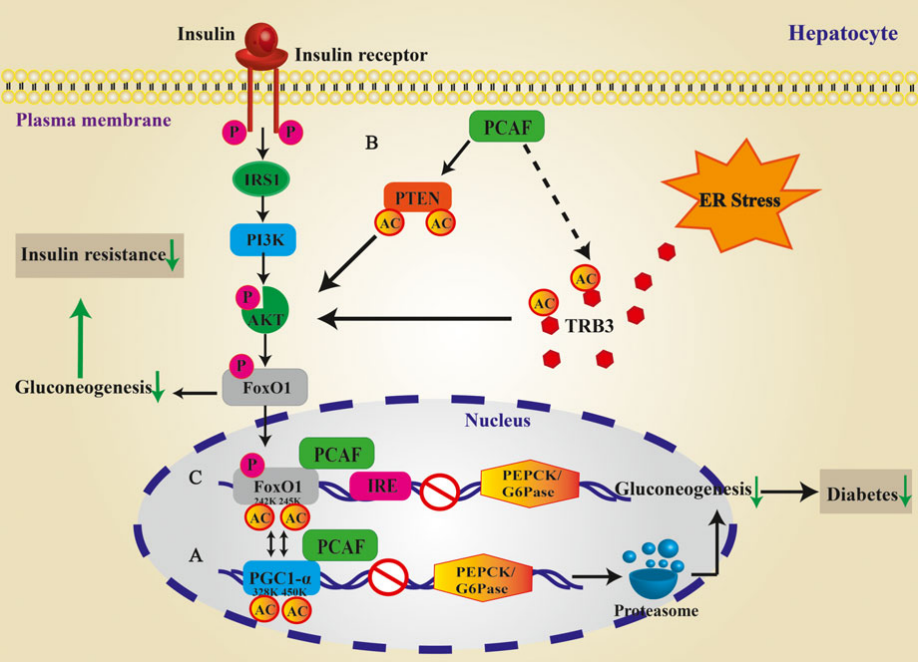
Potential contributions of PCAF‐induced acetylation to the development of hepatic Glucose homeostasis. A, PGC1‐α acetylation and hepatic gluconeogenesis. PCAF acetylate at lysine 328 and 450 residues, leading to its proteasomal degradation and attenuates PGC‐1α‐driven hepatic gluconeogenesis; (B) PI3K‐Akt/PKB signalling pathway and hepatic insulin resistance: ER stress activates TRB3; PTEN and TRB3 are both acetylated by PCAF increasing their activity to inhibit the PI3K‐Akt/PKB signalling pathway, therefore, leading to hepatic insulin resistance; (C) FoxO1 activity in diabetes. PCAF might regulate gluconeogenesis through binding and acetylating FoxO1 and decreasing both Pck1 and G6pc gene expression, resulting in decreased gluconeogenesis, therefore mitigating diabetes. PTEN, phosphatase and tensin homologue deleted on chromosome ten; TRB3, tribbles 3; Pck1, phosphoenolpyruvate carboxykinase; G6pc, glucose 6‐phosphatase

Accumulating evidence reveals that PGC‐1α increases the activity and increases the expression of many proteins involved in fatty acid β‐oxidation, the TCA cycle, and the electron transport chain, such as PPARα, and the glucocorticoid receptor.^76^, ^77^, ^78^ Notably, the adenoviral‐mediated expression of PGC‐1α in the hepatocytes strongly activates the entire programme of gluconeogenesis and increases glucose output.^79^ These results robustly demonstrate that PGC‐1α is a key transcriptional coactivator in hepatic gluconeogenesis, which is implicated in the onset of type‐2 diabetes. It was recently observed that PGC‐1α is acetylated and deacetylated by acetyltransferase GCN5 and deacetylase Sirt1, respectively, for regulation of its transcriptional activity in response to nutrients supply. When nutrient availability is high, PGC‐1α is acetylated by GCN5. As nutrients are exhausted, sirt1 activity is enhanced and PGC‐1α is deacetylated to maintain energy metabolism balance.^80^, ^81^ Intriguingly, another recent finding revealed that PCAF is a pivotal HAT that acetylates PGC‐1α in both fasting and diabetic states, and acetylates K328 and K450 residues in PGC‐1α, leading to its proteasomal degradation.^39^ It was demonstrated that adenoviral‐mediated expression of PCAF improves glucose homeostasis in obese mice by attenuating PGC‐1α‐driven hepatic gluconeogenesis: in an in vivo experiment liver‐specific knockdown of PCAF enhanced the transcriptional activity of PGC‐1α and stimulated hepatic gluconeogenesis^39^ (Figure 2A). These interesting results provide insight on the different roles for the two crucial HATs, GCN5 and PCAF, in acetylating PGC‐1α under physiological and pathological conditions, respectively, and enhance the current understanding of the acetylation of PGC‐1α in controlling glucose homeostasis in nutrient state and diabetes.

Given that the acetylation of FoxO1 at K242 and K245 by PCAF inhibits its transcriptional activity, it is likely that PCAF may modulate gluconeogenesis through additional mechanisms. Studies have shown that S‐phase kinase‐associated protein 2 (Skp2), which is involved in the regulation of glucose metabolism, interacts with ubiquitinases and promotes the degradation of FoxO1 through the Akt‐specific phosphorylation of Serine 256, thus suppressing the effects of this protein.^82^ This result suggests that the ubiquitination and degradation of FoxO1 by Skp2 may contribute to the onset of insulin resistance and diabetes. As the acetylation of FoxO1 by PCAF inhibits FoxO1‐induced transcription in the nucleus, there is also cooperation between PCAF and Skp2 in modulating FoxO1, thereby regulating glucose metabolism.^49^ Additionally, the genes involved in gluconeogenesis, including glucose 6‐phosphatase (G6pc) and phosphoenolpyruvate carboxykinase (Pck1), were recently demonstrated as target genes of FoxO1.^83^ In an in vivo experiment employing the adenoviral‐mediated transfer of a dominant negative FoxO1 into Lepr db/db mice, the reduction in nuclear FoxO1 decreased both Pck1 and G6pc gene expression, reducing gluconeogenesis and fasting blood glucose.^84^ Furthermore, antisense oligonucleotides of FoxO1 inhibit Pck1 and G6pc gene expression in primary hepatocytes which were stimulated with glucagons.^85^ These observations support the previous finding that FoxO1 increases hepatic gluconeogenesis through G6pc and Pck1 gene expression. Thus, PCAF might inhibit gluconeogenesis through additional mechanisms via the acetylation of FoxO1 (Figure 2C).

#### 2.2.2 PI3k‐Akt/Pkb signalling pathway and hepatic insulin resistance

The liver is a significant insulin‐sensitive organ in the regulation of glucose homeostasis.^86^, ^87^ Thus, insulin resistance in the liver was suggested as an underlying cause of metabolic syndrome, including hyperglycaemia, dyslipidaemia, and increased inflammatory factors.^87^ PI3‐kinase is a downstream target of insulin signalling, and its inhibition leads to hepatic insulin resistance. PI3‐kinase is mediated through the serine/threonine kinases, Akt/protein kinase B (PKB) and Protein kinase C, zeta (PKCζ), which are activated by PI3‐kinase via phosphoinositide‐dependent protein kinase‐1.^88^, ^89^, ^90^, ^91^ Notably, on the one hand, both Akt and PKCζ are under the inhibitory control of the lipid phosphatase and tensin homologue deleted on chromosome ten (PTEN), on the other hand, Akt is also inhibited by tribbles 3 (TRB3).^92^, ^93^ Moreover, Altomonte et al suggested that as a result of both the increased gene expression and the reduced acetylation of PTEN and TRB3 by PCAF, insulin stimulation of Akt and PKCζ is impaired along with increased expression of PTEN and TRB3 in rats and by the mechanisms leading to hepatic insulin resistance.^94^ This observation is consistent with another study showing that the acetylation of PTEN (Lys125 and Lys128) under the control of PCAF reduces its enzymatic activity.^95^ Moreover, TRB3 interacts with PCAF, but whether PCAF is a TRB3 acetylase remains unknown. Taken together, as PTEN and TRB3 inhibit the PI3K‐Akt/PKB signalling pathway, the acetylation status of PTEN and TRB3 and the balance between PCAF and HDAC activities also explain their role in insulin resistance and increased gluconeogenesis, which may indicate an involvement of PCAF‐induced acetylation and hepatic insulin resistance (Figure 2B).

## 3 PCAF IN LIVER INJURY AND MEDIATORS OF INFLAMMATION

Metabolism and immunity are closely linked. Long‐term hepatic metabolic disorders result in systemic low‐grade chronic inflammation of the liver. To a great extent, the inflammatory response contributes to the development of acute and chronic liver diseases.^96^, ^97^, ^98^ Recently, PCAF was demonstrated to influence various aspects of liver inflammation through multiple mechanisms, thus the targets of PCAF in proinflammatory reaction, oxidative stress, and liver injury will be discussed in this review.

### 3.1 Proinflammatory reaction and liver epithelial inflammatory response

**Figure 3:**
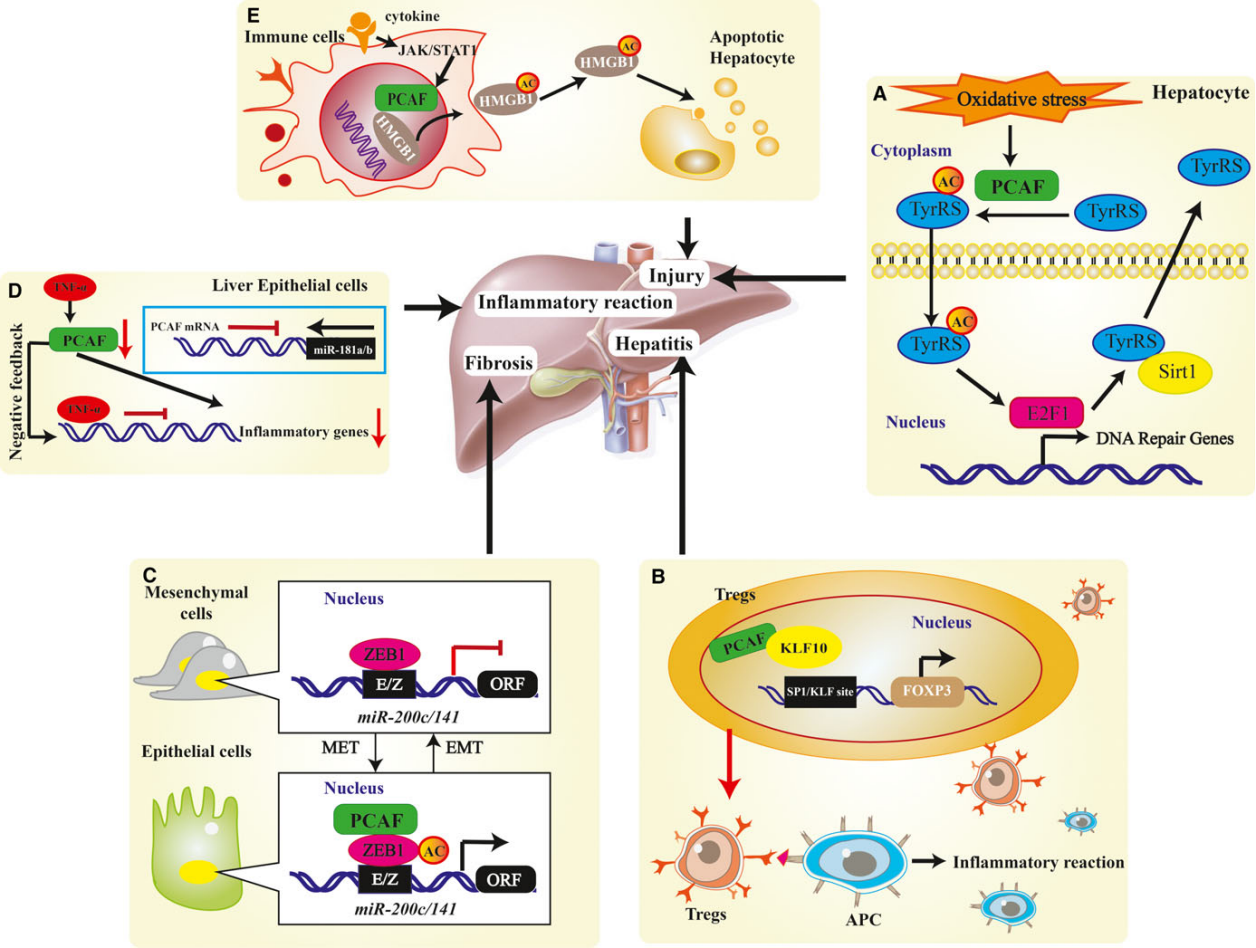
Potential contributions of PCAF‐induced acetylation to the development of liver injury and mediators of inflammation. A, Oxidative stress increases the level of PCAF; acetylation of TyrRS by PCAF promote the nuclear translocation of TyrRS, which may cause hepatocellular DNA damage under oxidative stress; (B) PCAF is recruited by KLF10 to the FOXP3 transcriptional regulatory regions that are critical for the induction of this gene which will amplify the chronic liver damage; (C) The EMT and MET events which are involved in liver fibrosis occurrence are regulated by PCAF acetylation on ZEB1; (D) As a target for miR‐181a/b, PCAF down‐regulation by TNF‐α provides negative feedback regulation to inflammatory reactions in liver epithelial cells responses, a process that may be relevant to the epigenetic fine‐tuning of epithelial inflammatory processes in general; (E) HMGB1 is acetylated by PCAF, this modification is associated with nonclassical vesicle‐mediated pathway which is an inflammatory response to liver injury. TyrRS, Tyrosyl‐tRNA synthetase; KLF10, transforming growth factorβ (TGFβ)‐inducible Kruppel‐like factor; FoxP3, forkhead box P3; Tregs, CD4^+^ ‐T cell; APC, antigen‐presenting cell; ZEB1, zinc finger E‐box‐binding homeobox 1, HMGB1, Serum high‐mobility group box 1; EMT, epithelial‐mesenchymal transition; MET, mesenchymal‐epithelial transition

Decades of research have confirmed that hepatocytes and cholangiocytes are involved in the initiation, regulation, and resolution of inflammatory reactions in the liver. Previous studies have shown that both cell types express receptors for tumour necrosis factor (TNF)‐α and the activation of downstream signalling cascades of TNF‐α receptors initiates a series of epithelial inflammatory reactions.^99^ Such hepatic epithelial cell responses are finely controlled under physiological conditions and reflect a delicate balance between effector functions and their potential to cause subsequent damage to liver tissues.^99^ Recently, Zhao et al reported that PCAF is a target for miR‐181a/b, play an important role in the regulation of inflammatory reactions in liver epithelial cells.^100^ A previous study established that the transcription of miRNA genes in cholangiocytes can be elaborately controlled through nuclear transcription factors associated with inflammation, such as NF‐κB.^101^ Functionally, miRNAs may activate epithelial inflammatory responses, including the production and release of cytokines/chemokines, the expression of adhesion and costimulatory molecules, and feedback regulation of epithelial homeostasis.^102^ Zhao et al showed that TNF‐α down‐regulates PCAF expression in liver epithelial cells, and cells pretreated with TNF‐α inhibited the transcription of inflammatory genes in response to subsequent TNF‐α stimulation. These authors further observed that overexpression of PCAF or inhibition of miR‐181a/b using anti‐miRs attenuated the inhibitory effects of TNF‐α pretreatment on epithelial inflammatory response to subsequent TNF‐α stimulation both in vitro and in vivo.^100^ Hence, fine‐tuning the inflammatory reactions in hepatocytes in response to TNF‐α stimulation may involve the miR‐181a/b‐mediated suppression of PCAF. Such a negative feedback regulatory loop may function in concert with other regulatory mechanisms to ensure finely controlled inflammatory responses in the liver. As a target for miR‐181a/b, PCAF down‐regulation by TNF‐α maintains a negative feedback regulation to inflammatory reactions in liver epithelial cell responses, a process that may be relevant to the epigenetic fine‐tuning of epithelial inflammatory processes in general (Figure 3D).

### 3.2 Liver inflammation and oxidative stress

Oxidative stress and liver inflammation/injury are strongly associated. It occurs as a consequence of imbalance between the production of reactive oxygen species and the body's ability to detoxify reactive intermediates.^103^, ^104^ More recently, Tyrosyl‐tRNA synthetase (TyrRS), a member of the 20‐enzyme family of aminoacyl‐tRNA synthetases, has been shown to translocate to the nucleus and protect against DNA damage because of oxidative stress, and the nuclear translocation of TyrRS is promoted by lysine acetylation in response to oxidative stress.^105^ In addition, PCAF and SIRT1 function as the acetyltransferase and deacetylase of TyrRS, respectively. Oxidative stress increases the level of PCAF and decreases the level of SIRT1 and deacetylase activity, all of which promote the nuclear translocation of hyperacetylated TyrRS. Furthermore, TyrRS is primarily acetylated on the K244 residue near the nuclear localization signal, and acetylation inhibits the aminoacylation activity of TyrRS.^105^ Notably, the acetylation of TyrRS by PCAF may cause hepatocellular DNA damage under oxidative stress, thus leading to immunoreaction in the liver (Figure 3A).

### 3.3 Liver injury and hepatic fibrosis

Drug‐induced liver injury (DILI) is a major cause of adverse drug reactions and is the leading cause of acute liver failure. Recently, Jonathan et al demonstrated that serum high‐mobility group box 1 (HMGB1) isoforms show promise as circulating biomarkers of hepatotoxin acetaminophen (APAP)‐induced DILI. HMGB1 is a nuclear nonhistone chromosomal protein that binds the DNA minor groove and is involved in DNA replication, repair, and energy homeostasis.^106^ Decades of research have shown that upon cell death, HMGB1 is released in a nonacetylated form, but in activated immune cells, HMGB1 is secreted in acetylated form.^107^, ^108^ Notably, HMGB1 is also acetylated by the HATs PCAF.^108^ PCAF‐induced acetylation of key lysine residues within the lysine‐rich NLSs of HMGB1 results in the cytoplasmic translocation of HMGB1 and directs HMGB1 for secretion via a nonclassical vesicle‐mediated pathway. Lea et al revealed the hyperacetylation of HMGB1 by PCAF in serum, suggesting that hepatotoxicity was associated with an inflammatory response.^106^ Thus, these authors showed histological evidence of DILI in APAP‐treated mice as a sensitive marker of APAP liver injury (Figure 3E), suggesting that serum HMGB1 isoforms, such as circulating biomarkers of hepatotoxin acetaminophen APAP‐induced DILI, are strongly correlated with the levels of HMGB1.

Similarly, many other factors show pronounced interactions with PCAF that are positively correlated with the intensity of liver injury and inflammation. A recent study in a chronic HBV hepatitis disease model demonstrated that the FOXP3 expression in the liver is associated with liver inflammation and is an underlying cause of tissue damage.^40^ Previous studies have established that the chronic liver damage is amplified by nonspecific liver‐infiltrating cells and CD4+ ‐T regulatory cell (Tregs) interaction pathways, T cell expression of programmed death 1, and inhibition of T effector cells through FOXP3+ Tregs are among the most powerful mechanisms for achieving a balanced immune response.^109^, ^110^ In addition, PCAF is recruited by transforming growth factor β‐inducible Kruppel‐like factor (KLF10) to FOXP3 in the transcriptional regulatory regions that are critical for the induction of this gene.^111^ Notably, Xiong and colleagues demonstrated that KLF10 possesses the dual capacity to either positively or negatively regulate FOXP3 via its differential association with PCAF or the histone deacetylase‐binding protein Sin3, respectively.^40^ The authors showed that in the absence of this Sin3‐binding event, KLF10 may couple with PCAF to facilitate the activation state of FOXP3. These studies suggested a novel mechanism of reversible chromatin‐dependent silencing of the key immunoregulatory gene FOXP3 through differential coupling of KLF10 to Sin3‐HDAC and PCAF (Figure 3B).

As discussed above, hepatic epithelial cell responses are finely controlled under physiological conditions and reflect a delicate balance between effector functions and their potential to cause subsequent damage to liver tissues.^99^ At present, research is focused on the mechanisms that potentially delay, and even reverse, the process of liver fibrosis. Accumulating evidence suggests that proliferating biliary epithelial cells directly respond via the epithelial‐mesenchymal transition (EMT) during the induction of fibrosis.^112^ The origin of fibrogenic cells in liver fibrosis remains controversial. As PCAF is a target for microRNA 181a/b, another member of miRNAs microRNA 200c/141 has also been associated with PCAF and to contribute to EMT.^113^ Recent studies have shown that the miR‐200 family and the transcriptional suppressor ZEB1 are important contributors to EMT. In addition, expression levels of miR‐200 family members and ZEB1/ZEB2 are closely and inversely associated. MiR‐200 down‐regulates the expression of the transcription factors ZEB1 and ZEB2 by binding to the 3′ untranslated region of the mRNA and preventing translation.^114^, ^115^ Mizuguchi et al showed that the acetyltransferases P300 and PCAF activate miR200c/141 transcription by interacting at its promoter region via the cysteine‐histidine‐rich (CH3) domain of P300.^113^ Furthermore, acetyltransferases overcome ZEB1 transcriptional suppression of miR200c, most likely through lysine acetylation of ZEB1, indicating a PCAF/P300 cooperative function in acetylating ZEB1, and leading to liver pathology^113^ (Figure 3C). These findings show a novel and unifying mechanism for the effect of acetyltransferases on miRNA transcription and the potential for morphological consequences. Moreover, this epigenetic mechanism will provide a potential therapeutic strategy to treat liver fibrosis.

## 4 PCAF IN LIVER CANCER AND TUMOUR GROWTH

An association between inflammation and cancer has long been suspected. Indeed, pathological changes in the metabolic and physiological status of the liver such as NAFLD, NASH, injury, and cirrhosis, result in the onset of liver cancer, including HCC.^116^, ^117^ Here, we review and discuss recent advances in the elucidation of cellular and molecular alterations, signalling pathways associated with PCAF, and their effects on hepatocarcinogenesis and tumour growth.

### 4.1 PCAF‐induced acetylation and HCC

**Figure 4:**
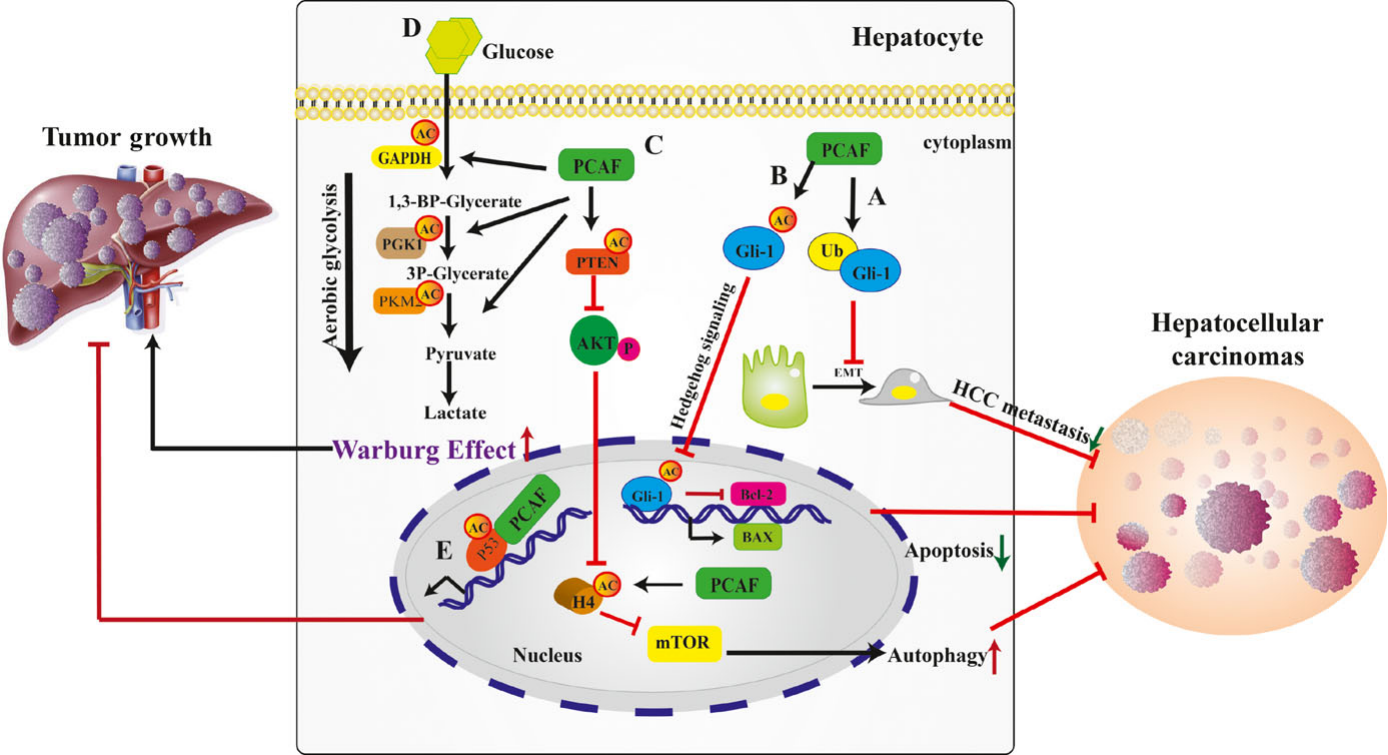
Potential contributions of PCAF‐induced acetylation to the development of hepatocellular carcinoma and liver cancer tumour growth. A, Gli1 is a direct negative target of PCAF‐induced EMT promoting tumour metastasis and invasions; (B) PCAF can directly acetylate cytoplasmic Gli1 proteinat, preventing its nuclear translocation and promoter occupancy, and consequently suppressing Hedgehog signalling in HCC; (C) PCAF acetylate PTEN at lysine 125 and 128 to inhibit PTEN regulation of PI3K/AKT signalling, therefore, inhibiting the PTEN‐regulated cell cycle arrest expression depends on the presence of growth factors, which results in the progression of HCC; (D) The three enzymes in the glycolysis pathway are acetylated by PCAF, accelerating aerobic glycolysis and increasing the Warburg effect in HCC; (E) P53 is acetylated by PCAF, and this modification increases the transcription of P53, a tumour suppressor, therefore inhibiting liver tumour growth. Gli1, glioma‐associated oncogene homologue‐1; Bcl‐2, B‐cell lymphoma‐2; BAX, Bcl‐2‐Associated X Protein; GAPDH, Glyceraldehyde‐3‐phosphate dehydrogenase; PKM2, pyruvate kinase M2; PGK1, Phosphoglycerate kinase 1; Ub, ubiquitination

As one of the end‐stage liver diseases, HCC ranks as the third leading cause of cancer mortality worldwide.^118^ Recently, with the rise of epigenetics, HCC metastasis and recurrence was demonstrated to involve lysine acetylation, especially, PCAF‐induced acetylation. Mounting evidence over the last 5 years implies different effects of PCAF on HCC. PCAF is likely expressed at low levels in most HCC cell lines. In addition, its overexpression induces HCC cell apoptosis and autophagy.^119^ PCAF has also been proposed to act as a metastasis suppressor of HCC by restraining the activity of transcription factor glioma‐associated oncogene homologue‐1 (Gli1), thus inhibiting the epithelial‐to‐mesenchymal transition (EMT) of HCC cells.^120^ EMT is controlled by a group of transcriptional repressors, including Gli1. Studies have suggested that the targeted inhibition of Gli1 promotes the growth inhibition of HCC with activated hedgehog signalling which has been observed in various human malignancies.^121^, ^122^ Li et al observed that PCAF was down‐regulated in HCC tissues compared with the adjacent nontumour tissues and significantly associated with malignant portal vein invasion. A functional study demonstrated that Gli1, as a direct negative target of PCAF, induced EMT and promoted tumour metastasis and invasions.^120^ As an antioncogene, PCAF works significantly in the development of HCC by suppressing HCC cell metastasis and EMT by targeting Gli1, indicating the value of PCAF for the suppression of HCC metastasis (Figure 4A). Moreover, PCAF can directly acetylate cytoplasmic Gli1 protein at lysine 518, preventing its nuclear translocation and promoter occupancy, consequently suppressing Hedgehog signalling in HCC. Furthermore, Gli1 can increase B‐cell lymphoma‐2 (Bcl‐2) expression and down‐regulate Bcl‐2‐Associated X Protein (BAX) while the forced expression of PCAF reduces Bcl‐2 expression, up‐regulates BAX and represses cell apoptosis.^123^ PCAF induces cell apoptosis by acetylating Gli1 and modulating a GLI1/Bcl‐2/BAX axis, which in turn suppresses HCC progression (Figure 4B). Thus, as a target of PCAF, Gli1 acts as a suppressor of HCC progression, and the two different binding effects of PCAF and Gli1 provide a new insight into the various target treatments of HCC.

It has been suggested that PCAF is linked with HCC through the regulation of autophagic processes. In vivo experiments have confirmed that PCAF‐induced autophagy inhibits tumour growth.^119^ Notably, subsequent in vitro experiments have shown that PCAF promotes autophagy by inhibiting Akt/mTOR signalling pathway, which was well studied in the past decades as a negative pathway of autophagy.^119^ In this study, PCAF expression inhibited the phosphorylation of Akt and mTOR kinase, whereas knockdown of PCAF stimulated Akt and mTOR kinase activity. These results demonstrated that PCAF down‐regulates Akt/mTOR signalling pathway to activate autophagy (in addition to apoptosis) and mediate cancer cell death. Similarly, PCAF affects HCC cell apoptosis through acetylating histone H4 and inactivating AKT signalling. PCAF overexpression induced cell apoptosis and growth arrest with the increased Histone H4 acetylation and inactivation of AKT signalling in Huh7 and HepG2 cells. Moreover, coimmunoprecipitation assay confirmed that PCAF protein functions as a HCC repressor, binding to histone H4 protein in the nucleus of Hep3B cells, and eventually promoting cell apoptosis and functioning as a HCC repressor^124^ (Figure 4C). Establishing that PCAF promotes autophagy in HCC makes this novel molecular mechanism an attractive therapeutic strategy of HCC treatment.

Given that PTEN protein can be acetylated by PCAF, this mechanism can also contribute to the occurrence of HCC.^90^ The tumour suppressor activity of PTEN principally exerts its antagonistic effects on the antiapoptotic, proliferative, and hypertrophic activity of PI3K.^125^ In cancer biology, PTEN is frequently mutated or deleted in a wide variety of tumours. In patients with HCC, mutations, decreased promoter activity, and decreased PTEN expression are common occurrences.^126^ Indeed, the expression of PCAF increases the acetylation of lysine residues (Lys125 and 128) within the catalytic cleft of PTEN.^90^ The acetylation of PTEN caused by PCAF was directly associated with the inhibition of PTEN regulation of PI3K signalling, and the inhibition of PTEN‐regulated cell cycle arrest expression depends on the presence of growth factors, resulting in the progression of HCC.^90^ As inflammation, EMT, and genomic alterations are typical features of HCC, impaired PTEN expression or activity can thus represent an important step in progression of NAFLD towards understanding HCC.^127^, ^128^ These results indicate that a PTEN acetylation mechanism supports a correlation between distinct cancer‐relevant pathways that are essential to the control of growth factor signalling and gene expression (Figure 4C).

### 4.2 Warburg effect and tumour growth

Almost all cancer cells mainly rely on an aerobic glycolysis pathway to utilize glucose, the so‐called “Warburg Effect” first described by Dr. Otto Warburg.^129^, ^130^ The two enzymes that control the production of ATP during aerobic glycolysis in cancer cells are PGK1 and pyruvate kinase M2 (PKM2). Both enzymes were acetylated by PCAF.^37^, ^131^ Using immunoprecipitation of endogenous proteins and in vitro pull‐down assay with recombinant, a study identified and validated that PCAF as the acetyltransferase acetylating PGK1 K323 acetylation in liver cancer, this modification is characterized as an important positive regulation of PGK1 enzymatic activity and its oncogenic function.^37^ In addition, they further identified and validated that SIRT7 is the deacetylase of PGK1 at K323. And it can be proposed that PCAF‐induced K323 acetylation of PGK1 enhances its activity and promotes cancer cell proliferation and tumorigenesis (Figure 4D).

Similarly, the enzyme PKM2 was also associated with PCAF.^131^ The K305 site of PKM2 can be acetylated by PCAF, enhancing its association with the heat shock cognate protein 70 (HSC70) thereby promoting its lysosomal‐dependent molecular chaperone‐mediated autophagy process of degradation.^131^ High‐glucose concentrations increased the acetylation of PKM2 at Lys305, decreased PKM2 abundance, and increased association of PKM2 with the acetyltransferase PCAF. Ectopic expression of PCAF increased acetylation of PKM2 at K305 and decreased PKM2 activity. The glucose‐induced acetylation of PKM2 by PCAF, which inhibits its activity and triggers its autophagic degradation, promotes increased amounts of glycolytic intermediates that enable tumour growth and cell proliferation (Figure 4D).

Glyceraldehyde‐3‐phosphate dehydrogenase (GAPDH), a housekeeping glycolytic enzyme, has also shown to be acetylated by PCAF.^132^ Lysing acetylation activates GAPDH, which will alter along with the dynamic changes of external environment, such as glucose concentration. A recent study demonstrated that GAPDH K254 was acetylated by PCAF and deacetylated by HDAC5. Acetylation of GAPDH by PCAF promoted cell proliferation and tumour growth^132^ Most enzymes in glycolysis, including GAPDH, are acetylated in the liver, suggesting a positive role for PCAF‐induced acetylation of GAPDH in liver tumour growth and providing a potential therapy target for cancer. Furthermore, K117 and K251 are the putative GAPDH residues that could also be acetylated by PCAF. These modifications enable the entry of GAPDH into the nucleus for gene transcription and DNA repair^133^ (Figure 4D).

Additionally, as a tumour suppressor, p53 has also shown to be acetylated by PCAF in response to DNA damage.^41^ CBP/p300 acetylates p53 to achieve full transcriptional activity. This mechanism raised the question of whether PCAF can also acetylate p53 and if so, whether the substrate specificity would be distinct from that of CBP/p300. To address this question, an in vitro study suggested that PCAF and p300 acetylated full‐length p53 (p53 FL), and both enzymes had nearly equal acetylation activities. Lysine 320 in p53 is acetylated by PCAF, and PCAF acetylation activates the sequence‐specific DNA binding of p53. The acetylation site at K320 is targeted by PCAF and the acetylation at K373 is targeted by p300. Both p300 and PCAF increase the affinity of p53 to bind its cognate DNA site.^41^ These findings suggested that p53 PCAF‐induced acetylation is of great importance for DNA damage and cancer (Figure 4E). As p53 serves as a tumour suppressor protein it might have significant connection to liver cancer. This mechanism could provide us with a new pathway for the treatment of liver cancer.

## 5 CONCLUDING DISCUSSION AND PERSPECTIVE

The development of PTMs in liver disease prompted considerable research interest on novel perspectives in the past 20 years. As one of the PTMs, lysine acetylation has been associated with immunological and metabolic pathways. Most recent studies have started to shed light on the roles of different HATs in diverse biological functions. Intriguingly, similar to other HATs, PCAF is now emerging as a regulator of diverse nutrition metabolism functions and diseases, especially in the liver. Studies using improved methods to optimize the detection of substrate‐specific acetylation suggest that PCAF, through acetylation of complex interactive network substrates, enables a wide range of interactions with components of important metabolic and immunological pathways in the liver, such as lipogenesis, gluconeogenesis, insulin sensitivity, the PI3K‐Akt/PKB signalling pathway, and epithelial inflammatory response, among others. These interactions are closely associated with the main liver diseases including hepatogenous diabetes, NALFD, liver fibrosis, and cancer (Table 1).

**Table 1:** Overview of the fine‐tuning mechanism of PCAF in liver diseases via the regulation of different targets

Disease	Activity	Targets	Effect	Metabolic response	Reference
Liver cancer	Ubiquitination	Gli‐1	Inhibitory	HCC metastasis↓	120
	Acetylation	PGK1	Stimulatory	Cell proliferation and tumorigenesis↑	37
	Acetylation	PKM2	Inhibitory	Cell proliferation and tumorigenesis↑	131
	Acetylation	GAPDH	Stimulatory	Cell proliferation and tumorigenesis↑;DNA repair process↑	132, 133
	Acetylation	Histone H4/AKT signalling	Inhibitory	Cell apoptosis↑ and HCC↓	124
	Acetylation	Gli‐1	Inhibitory	GLI1/BCL2/BAX axis↓ and HCC↓	123
	Induction	AKT/mTOR	Inhibitory	Autophagy↑ and HCC↓	119
	Acetylation	PTEN	Inhibitory	Tumorigenesis↑ and HCC↑	90, 127, 128
	Acetylation	P53	Stimulatory	Apoptosis↓	41
Hepatic metabolic syndrome	Acetylation	CREBH	Stimulatory	Fasting‐induced hepatic steatosis and hyperlipidaemia↓	71
	Acetylation	USF‐1	Stimulatory	Lipogenesis↑	55
	Acetylation	ACLY	Inhibitory	Lipogenesis↑	38
	Binding/Acetylation	FoxO1	Inhibitory	FFA oxidation↑ TG synthesis↓	49
	Interaction	XBP‐1S	Stimulatory	Hepatic steatosis↑	60
	Acetylation	PGC1‐α	Inhibitory	Glucose production↓ Insulin sensitivity↑ obesity and diabetes↓	39
	Acetylation	PTEN and TRB3	Inhibitory	Gluconeogenesis↓ Insulin resistance↓	89, 90
	Acetylation	FoxO1	Inhibitory	Gluconeogenesis↓ diabetes↓	49
Immune disease	Acetylation	TyrRS	Inhibitory	Repair pathways for damaged DNA↓	105
	Binding	miR‐181a/b	Inhibitory	Proinflammatory Genes↓	100
	Induction	FOXP3	Stimulatory	Immune Activation↑	40
	Acetylation	miR‐200c/141 (ZEB1)	Stimulatory	Liver fibrosis↑	113
	Acetylation	HMGB1	Stimulatory	Hepatocyte apoptosis↑	106, 108

To date, the molecular events by which PCAF exerts a protective role at the hepatic organelle level, regulating the hyperglycaemia, ER stress to oxidative stress, and Warburg effect are still not fully understood. However, based on this review, it is increasingly clear that PCAF fine‐tunes the progress of liver diseases ranging from metabolic syndrome to inflammatory disease and tumour growth. Common to other HATs, PCAF not only acts on liver cellular targets for metabolism regulation including p53, FoxO1, PGC1‐α, ACLY, PTEN, CREBH, but also shows differences among the other HATs in their roles on diseases. For example, PCAF acetylates PGC1‐α and decreases gluconeogenesis in hepatogenous diabetes, while another HATs GCN5, exerts its effect only in response to nutrition alterations. In addition, PCAF exerts its different effects from other HATs on regulating liver injury, fibrosis, and especially HCC. For example, on one hand, PCAF acts as a suppressor of HCC progression by acetylating Gli1, histone H4, and PTEN. On the other hand, PCAF also acetylates PGK1, PKM2, and GAPDH to induce a Warburg effect in liver tumour growth. This contradictory phenomenon suggests the value of PCAF for research on liver diseases.

The discovery of novel functions for PCAF‐mediated acetylation of transcription factors and enzymes on liver diseases opened new avenues of research into these less‐understood modifications. It also raised several important questions that may be crucial for assessing the potential specificity and efficacy of PCAF modulation as a therapeutic strategy of liver diseases:

1. Given the importance of PCAF in the occurrence of Warburg effect, will the down‐regulation, a general phenomenon during diseases, and inactivation of PCAF in such contexts ease the degenerative changes in aerobic glycolysis and cancer?
2. How does the interaction between the pleiotropic effects of PCAF on impact tumour biology, and which of these mechanisms are most relevant in the occurrence of cancer?
3. PCAF shows either positive or negative effects on the regulation of different sites in hepatic steatosis: which is the dominating function?
4. Can modified targets of PCAF in different stages of liver diseases at the same site act as the biomarkers or therapeutic targets of different liver diseases, and how do we balance the interaction between PCAF and HDACs (eg SIRTs) in the liver?

## CONFLICT OF INTEREST

The authors declare that they have no conflict of interest.
